# Ion-mediated conformational switches

**DOI:** 10.1039/c4sc03525a

**Published:** 2014-11-21

**Authors:** Peter C. Knipe, Sam Thompson, Andrew D. Hamilton

**Affiliations:** a Department of Chemistry , Chemistry Research Laboratory , University of Oxford , 12 Mansfield Road , Oxford OX1 3TA , UK . Email: sam.thompson@chem.ox.ac.uk ; Email: andrew.hamilton@chem.ox.ac.uk ; Tel: +44 (0)1865 275978

## Abstract

Molecular switches are ubiquitous in Nature and provide the basis of many forms of transport and signalling. *Single* synthetic molecules that change conformation, and thus function, reversibly in a stimulus-dependent manner are of great interest not only to chemists but society in general; myriad applications exist in storage, display, sensing and medicine. Here we describe recent developments in the area of *ion*-mediated switching.

## Abstract 2

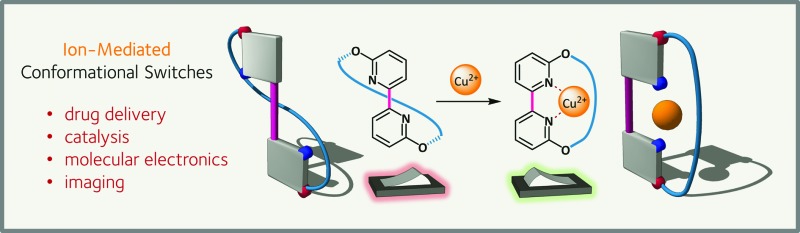
The ability to control the conformation of a single molecule in a reversible and stimulus dependent manner holds great promise for fields as disparate as drug delivery and molecular electronics. Here we offer a perspective on recent developments in ion-mediated switching architectures and their ability to perform in a range of settings.

## Introduction

The construction of single molecules that change conformation predictably in response to an external stimulus presents an opportunity to exert control over a sub-nanometer scale world.^1^ Chemists have long sought to replicate the high affinities and exquisite selectivities of natural systems in which precisely folded proteins frequently provide a scaffold for switching. While peptidic systems typically involve the complex interplay of a large number of stabilizing interactions, the bottom-up approach of a synthetic chemist allows tuning of architecturally minimal designs to provide an output in response to a specific stimulus. The last decade has seen an explosion of interest in this area with the development of switches responsive to light,^2,3^ redox processes,^4^ and the focus of this Perspective – ions. Potential applications of molecular switches include bio-imaging,^5^ drug delivery,^6^ organic light-emitting diodes,^7^ molecular electronics,^8^ catalysis,^9^ and receptors for use in a range of settings.^10^

Equally important is the insight that synthetic switches can give into the fundamentals of molecular recognition – especially those in physiological environments. Protein folding, membrane transport, viral entry and translocation, numerous post-translational modification processes and the regulation of epigenetics are controlled at the molecular level by recognition events that are triggered by subtle conformational ‘switches’.^11^ These may be non-covalent or covalent, but are frequently reversible, rendering functionally ‘on’ and ‘off’ conformational states. Elucidation of the conformationally determinant interactions in such cases is frequently hampered by difficulty in identifying top-down strategies that provide sufficient decoupling of variables. From a physical organic perspective, model systems allow the problem to be addressed with simple, readily tuneable systems. Whilst molecular switches are generally assumed to alternate between two states upon the application of a stimulus, a related class of molecules – the torsion balance^12,13^ – provides a means of measuring intra- and intermolecular forces through comparison of different equilibrium positions. The insights gained are not only useful for rationalisation of conformational behaviour, but can be fed into a subsequent design phase.

In seeking to exploit the potential of molecular switching to perform macroscopic level work, the last few years have seen strides made towards the development of molecular machines – systems in which work is done against a load.^14–17^ Light and redox processes have proven popular energy inputs for these systems, while the use of ion gradients to power motors has been less well studied.^3,18^

The field of molecular switching is now well established, with individual architectures and their stimuli becoming the focus of numerous research and review articles. Here we examine single molecule conformational switches that are mediated by their interaction with anions and cations. Elegant examples of mechanically interlocked ion-responsive switching systems such as rotaxanes and catenanes have been reviewed extensively elsewhere.^19–23^

## Nature as a source of inspiration

**Scheme 1:**
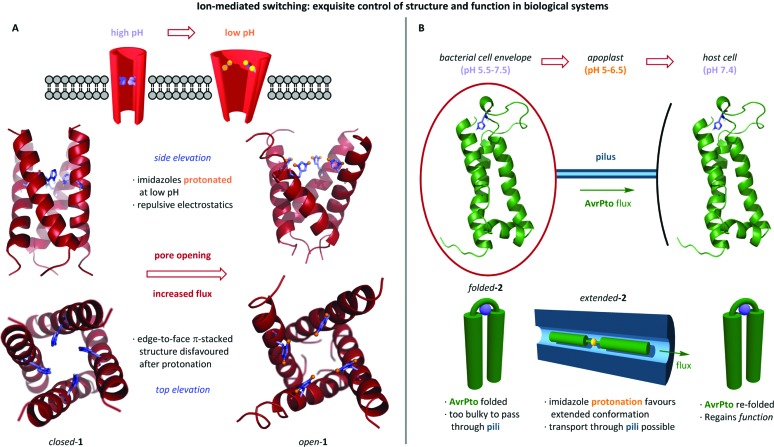
(A) The acid-mediated opening of the viral M2TM trans-membrane protein **1** – an impressive natural molecular switch. (B) pH-dependent unfolding of bacterial protein AvrPto **2** allows its passage into the host cell *via* the pili, the small aperture of which does not allow passage of the folded protein. The key histidine residue H87 is illustrated.

Nature has evolved exquisite proton-mediated conformational switches which are frequently found in the vicinity of cell membranes where trans-membrane pH gradients are exploited to imbue biomacromolecules with tuneable function through conformational change. The role of pH in regulating the activity of trans-membrane protein channels has been studied in the M2 protein of influenza viruses.^24^ Solid-state NMR revealed the structure of the M2 trans-membrane (M2TM) domain in a lipid membrane between pH 8.4 and 4.5. Switching was observed between a closed state (Scheme 1A, *closed*-**1**) at high pH in which the central pore is blocked by edge-face π-stacked histidines, and an open state (*open*-**1**) at low pH in which protonation of the histidines leads to dilation of the pore. It is postulated that in the closed state, the tightly packed imidazoles prevent formation of an H-bonded water chain, hence inhibiting trans-membrane proton transfer. However, protonation at low pH results in repulsion between the imidazolium rings, causing pore widening and allowing proton transfer to proceed.

Changes in pH can also induce conformational change in the *substrate* of trans-membrane transportation. Nicholson *et al.* investigated the behaviour of bacterial effector protein AvrPto **2**, a helical bundle known to pass *via* the bacterial type III secretion system and through the pili whose small internal diameter necessitate unfolding of the native AvrPto (Scheme 1B).^25^ Unfolding is facilitated by the pH gradient between the bacterial cytoplasm, apoplast and host cell cytoplasm. It was postulated that protonation of key histidine residue his87 disfavours the folded state, such that upon approaching the acidic environment of the apoplast AvrPto adopts an extended conformation that allows it to traverse the bacterial membrane and enter the host cell through the narrow pilus.

## Cation binding

**Scheme 2:**
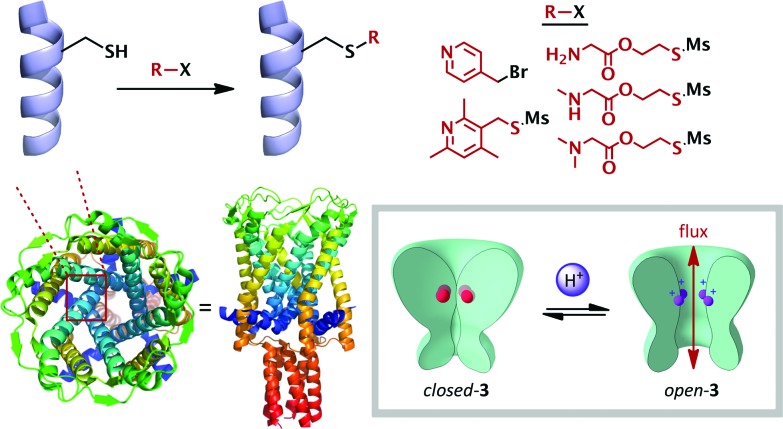
Rationally-modified membrane proteins (**3**) as pH-switchable ion gates. Crystal structure (PDB: ; 2OAR) of the homologous protein expressed by *Mycobacterium tuberculosis*.

**Scheme 3:**
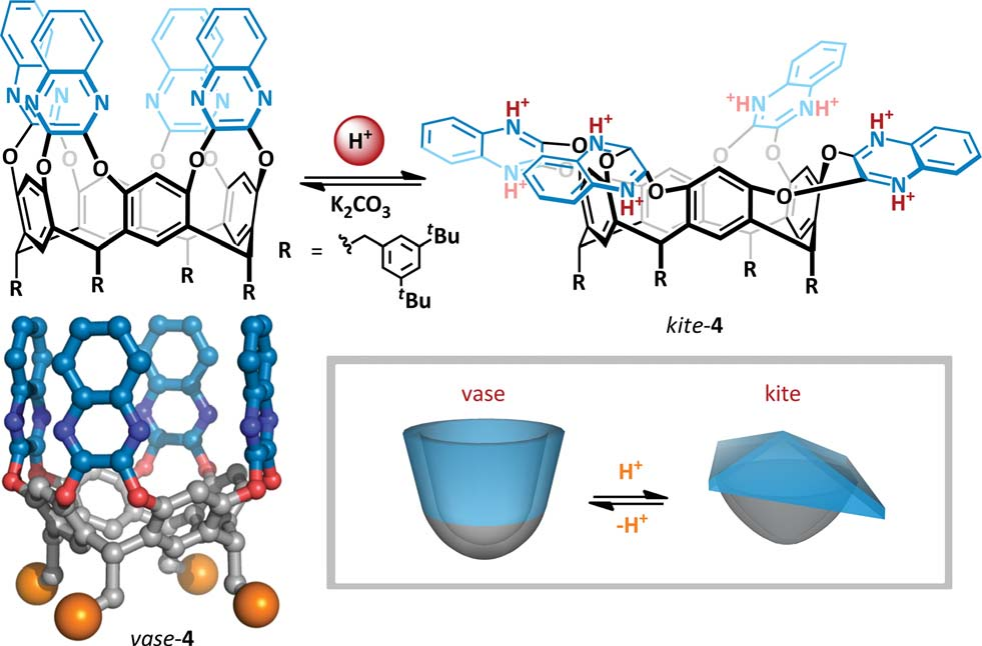
Acid mediated interconversion between vase and kite cryptands **4**. The authors did not investigate the precise extent of protonation; the fully protonated kite is illustrated. Crystal structure of cryptand **4** (R-groups are represented as orange spheres).

The simplest and most widely studied ionic stimulus for conformational change is protonation. Chemists have sought to emulate the exquisite pH-responsive behaviour exhibited by Nature. The endogenous mechanosensitive channel with very large conductance (MscL) is a homopentameric protein which governs nonselective ion transport in response to mechanical stress in *E. coli*.^26^ Kung has elegantly shown that mutant MscL proteins with hydrophilic residues substituted for gly22 showed increased ionic flux through the pore,^27^ and that cysteine mutants at this position functionalized with charged substituents caused the pore to spontaneously open.^28^ Feringa and Meijberg developed this concept into a rationally-designed pH-responsive bacterial channel protein **3**, exhibiting analogous behaviour to the M2TM membrane protein described above (Schemes 1 and 3).^29^ A range of amine side-chains were introduced onto the cysteine mutant, and were chosen with a variety of p*K*_aH_ values. These functionalized proteins were then incorporated into a liposomal wall, and their gating capacity was measured by the efflux of a fluorescent reporter. The release of the reporter was found to be highly pH dependent, with low levels at pH values above the p*K*_aH_ of the side-chains (5–10% after 30 min), and much greater efflux below this value (up to *ca.* 45%). This behaviour is likely due to increased electrostatic repulsion between the five cationic ammonium species leading to an increase in the internal diameter of the pore. Since increased hydrophobicity has been shown to induce pore closure,^27^ this also acted as a modulating factor on the switching behaviour, and allowed further tuning of the switch to access the pH range observed in the vicinity of solid tumours,^30^ demonstrating proof-of-principle for switch-mediated targeted drug delivery (Scheme 2).

The resorcin[4]arene cavitands (**4**) introduced by Cram^31^ have been investigated as a potential scaffold for the implementation of cation-mediated switching.^32–35^ In the absence of stimuli, these cavitands exist in two interconverting ‘vase’ and ‘kite’ forms. The kite form is favoured at low temperatures (<213 K), but upon warming (to >318 K) the bowl conformation predominates since the entropic penalty for solvation of the larger kite becomes too great. Diederich showed that upon addition of TFA to cryptand **4** at room temperature, the interconverting vase-kite mixture was converted exclusively to the kite form (Scheme 3). ^1^H NMR titration analysis of the characteristic benzylic methine residue exhibited a 2 ppm upfield shift consistent with formation of the kite, and UV/VIS spectroscopy confirmed the structure formed was comparable to the low temperature kite conformation. This behaviour was rationalized on the basis of protonation of the quinoxalines resulting in dipolar repulsion between the cationic arms of the cavitand. Since host molecules can be incorporated into the cavity,^36^ such switchable containers offer the promise of targeted delivery of molecular cargo.

**Scheme 4:**
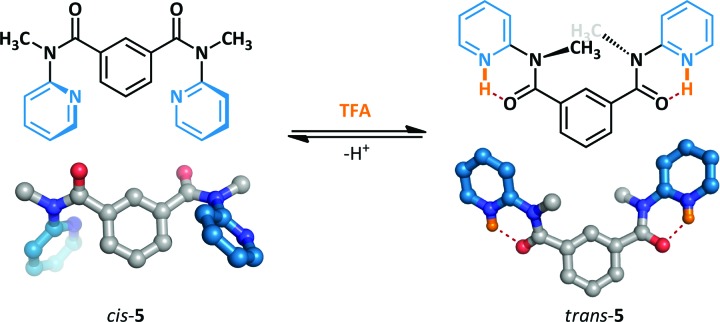
Acid-mediated switching between amide conformers.

The acid-mediated switching behaviour of a series of *N*-methyl-*N*-(2-pyridyl)benzamides has been investigated by Okamoto (Scheme 4).^37,38^ Prior to the addition of the acid switching stimulus, **5** adopts primarily the *cis*-amide conformation. The acid-promoted switching behaviour was examined in the solution and solid state by ^1^H NMR spectroscopy and X-ray crystallography respectively, and the amides were demonstrated to undergo conformational change to the *trans*-amide. The protonated pyridinium ions form stabilizing N–H···O hydrogen bonds to the adjacent amides which are not accessible to the *cis*-conformer; this enforces coplanarity of the amide and pyridine, leading to the overall conformational change observed.

**Scheme 5:**
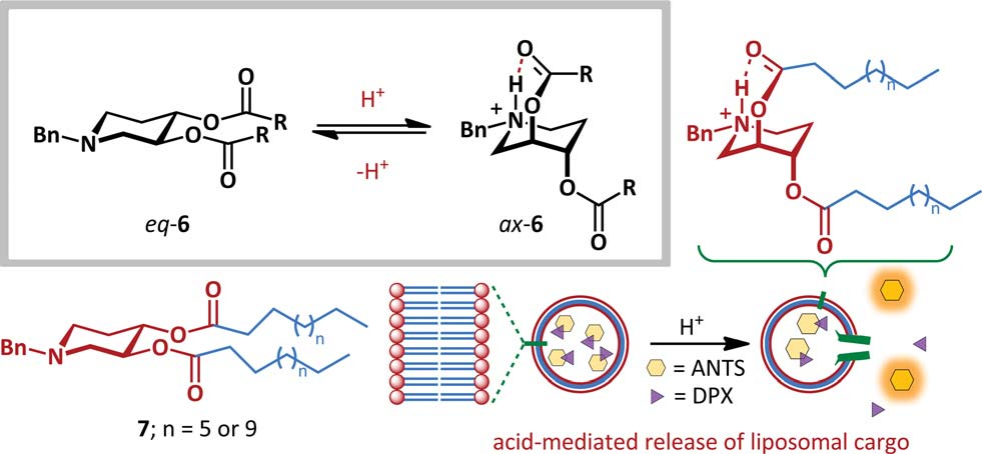
Samoshin's approach to acid-mediated ring flipping, and application to pH-dependent delivery of liposomal cargo.

The fast and reversible nature of ring-flipping within cyclohexanes and related structures makes them well-suited to molecular switching applications. Significant research has been carried out under this premise.^39–44^ For example, Samoshin has exploited the pH-dependent conformational switching of piperidine derivative **6** (Scheme 5).^43^ At neutral pH the preferred conformation places all substituents in equatorial positions, as determined by application of Eliel's equation to solution-state NMR data,^45^ and by DFT analysis. Upon protonation of the basic piperidine, the scaffold undergoes ring-flipping to project the ester side-chains axially with up to 72% preference. This conformational change allows stabilization of the protonated amine by formation of a transannular intramolecular hydrogen bond. This approach was demonstrated to be a viable strategy for one of the most promising potential applications of molecular switches: targeted drug delivery. When switch **7** was incorporated as a component of a liposomal bilayer encapsulating a fluorescent dye (8-aminonaphthalene-1,3,6-trisulfonic acid, ANTS) and quencher (*p*-xylene bis(*N*-pyridinium bromide), DPX), no leakage of the dye was observed at pH 7.4. However, upon acidification to pH 4, up to 32% leakage was attained, with the increased distance between ANTS and DPX leading to greater fluorescence. This behaviour is attributed to switching of **7** within the liposome reducing the efficiency of bilayer packing, since the effective hydrophobic tail length is reduced, and the size and charge of the head-group significantly altered.

**Scheme 6:**
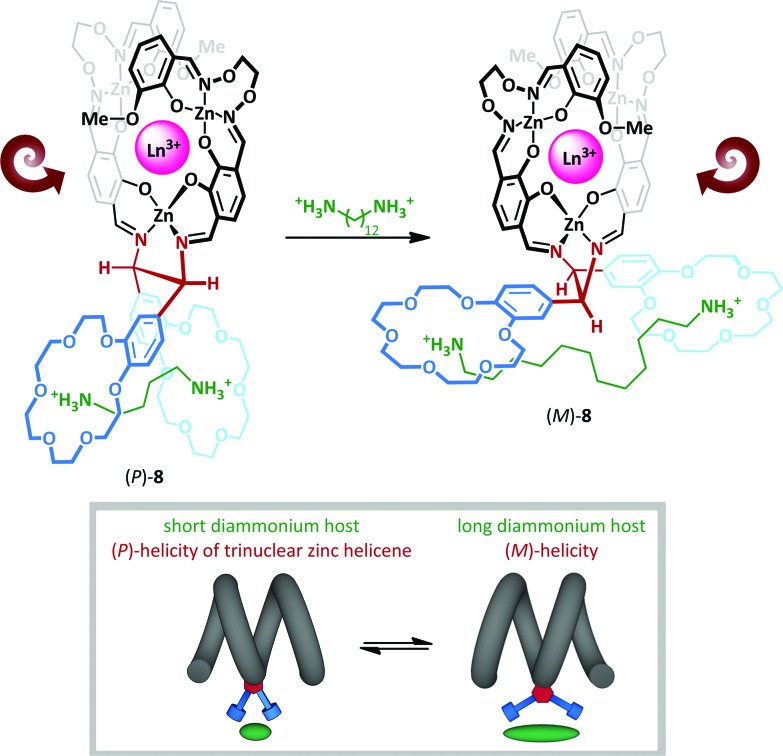
Diammonium cation chain length mediates switching between helical diastereoisomers of **8**.

The interchange between *anti* and *gauche* conformations has also been exploited by Akine and Nabeshima,^46^ who have described a cation-dependent helical inversion switch (Scheme 6). Trinuclear zinc(ii) lanthanum(iii) helicene complex **8** adopts a (*P*)-helical form in the presence of short-chain diammonium cation, which is complexed by both 18-crown-6 macrocycles in a *gauche* arrangement about the ethylenediamine linkage. Upon replacing the short diammonium cation with a much longer one, the 18-crown-6 ‘levers’ are forced further apart, ultimately adopting an *anti*-conformation; this enforces a negative N–C–C–N torsion angle, which subsequently leads to stabilization of the (*M*)-helical form. The authors describe the chiral diamine motif as a molecular ‘transducer’, communicating the length of the diammonium cation into a helical screw-sense readout.

**Scheme 7:**
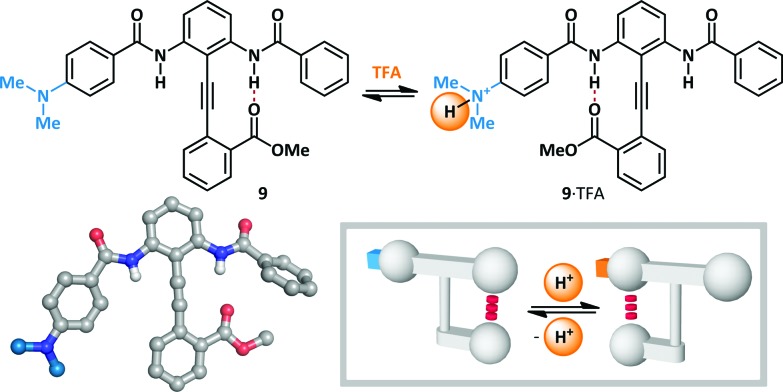
The diphenylacetylene platform for acid-mediated molecular switching.

Hamilton has modified Kemp's β-turn mimic^47–52^ to give a series of bisbenzamido-diphenylacetylenes that display an equilibrium between two hydrogen-bonded states (Scheme 7).^53^ Since the position of the equilibrium is determined by the relative strength of the two *meta*-positioned hydrogen bond donors, the conformation is rendered switchable by the modulation of their Brønsted acidity. In the absence of an acid stimulus, diphenylacetylene **9** favours hydrogen bonding primarily to the unsubstituted benzamide (4 : 3). Treatment with TFA (6 eq.) led to complete switching towards the *para*-dimethylaminobenzamide (99 : 1), since protonation of the basic amine leads to increased acidity of the corresponding amide. This constitutes a rare example in which protonation at a position *remote* to the switching motif provides the necessary stimulus for switching, and where the added proton itself does not participate in conformationally-determinant hydrogen bonding. Molecular switches have subsequently been developed based upon the diphenylacetylene platform that are responsive to stimuli including Lewis and Brønsted acids, Brønsted bases and changes in redox potential.^4,54,55^

**Scheme 8:**
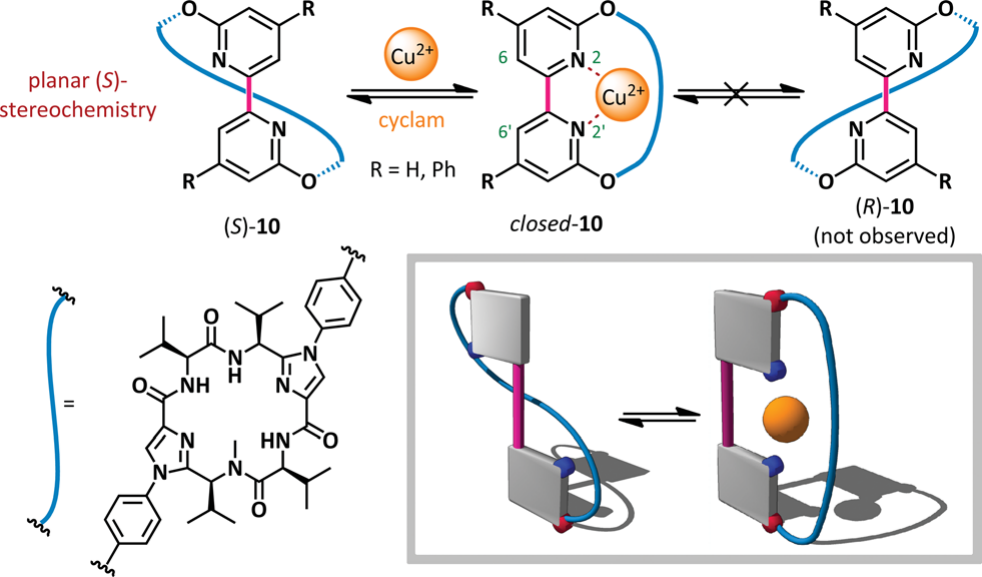
A 2,2′-bipyridine-based molecular hinge **10**, responsive to Cu^2+^ ions as stimuli.

Haberhauer has reported a copper(ii)-mediated ‘molecular hinge’ based on a 2,2′-bipyridine core (**10**, Scheme 8).^56^ Steric repulsion between protons at the 6- and 6′-positions, coupled with lone pair repulsion between the nitrogen atoms, ensured that, in the absence of external stimuli, the hinge adopted an open conformation with an N–C–C–N dihedral angle close to 180°. A chiral peptidic intramolecular tether was introduced *via* substitution at the 3- and 3′-positions, and was shown to induce formation of solely the planar diastereoisomer (*S*)-**10**. The homochirality of the tether was the determinant of this conformational preference and computation indicated that the (*R*)-diastereomer was up to 42 kJ mol^–1^ (R = Ph) higher in energy than the (*S*)-conformer. Upon the addition of Cu(OTf)_2_, complexation of the bipyridine with copper(ii) was monitored by CD spectroscopy. The positive and negative Cotton effects observed for planar chiral conformer (*S*)-**10** at 272 and 293 nm respectively disappeared completely, consistent with formation of *closed*-**10** which does not possess planar chirality. The reversibility of the switching was also demonstrated: addition of cyclam to complex the copper caused complete recovery in the CD spectrum, consistent with reversion to the open conformer (*S*)-**10**.

**Scheme 9:**
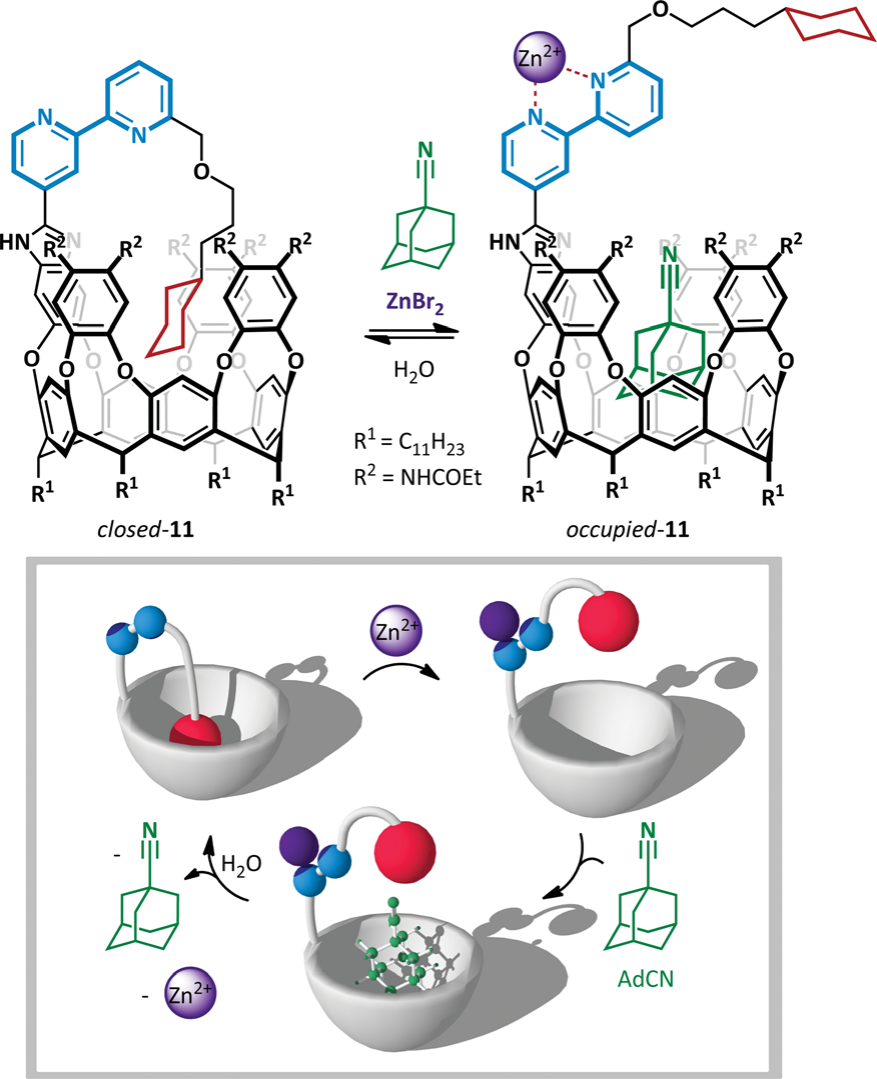
A zinc(ii)-ion switchable cavitand **11**.

Rebek has also used the chelating power of 2,2′-bipyridines to allow zinc(ii)-mediated switching of cavitand **11**,^57^ the unique conformation of which was likened to the *Ouroboros* – the ancient symbol of a serpent devouring its own tail (Scheme 9).^58^ When bipyridine **11** is not coordinated, lone pair and steric repulsion favour the *anti*-conformation, positioning the tethered guest cyclohexane in the vicinity of the cavitand and favouring the formation of an intramolecular inclusion complex. When this complex was treated with a potential intermolecular guest, 1-adamantane-carbonitrile (AdCN), no exchange was observed, indicating that the intramolecular ‘autophagic’ arrangement is highly favoured. However, upon treatment with AdCN and ZnBr_2_ in acetonitrile complete switching occurs to the opened form of the cavitand, and 45% occupation of the cavitand by AdCN is observed (with the remainder occupied by solvent). This behaviour is a consequence of the conformational switching of the 2,2′-bipyridine to the *syn*-arrangement, with concomitant loss of the intramolecular inclusion interaction, leaving the host cavitand unoccupied and available to accommodate alternative guests. Addition of water washed out the zinc(ii), causing ejection of the intermolecular hosts by the more favourable tethered cyclohexane.

**Scheme 10:**
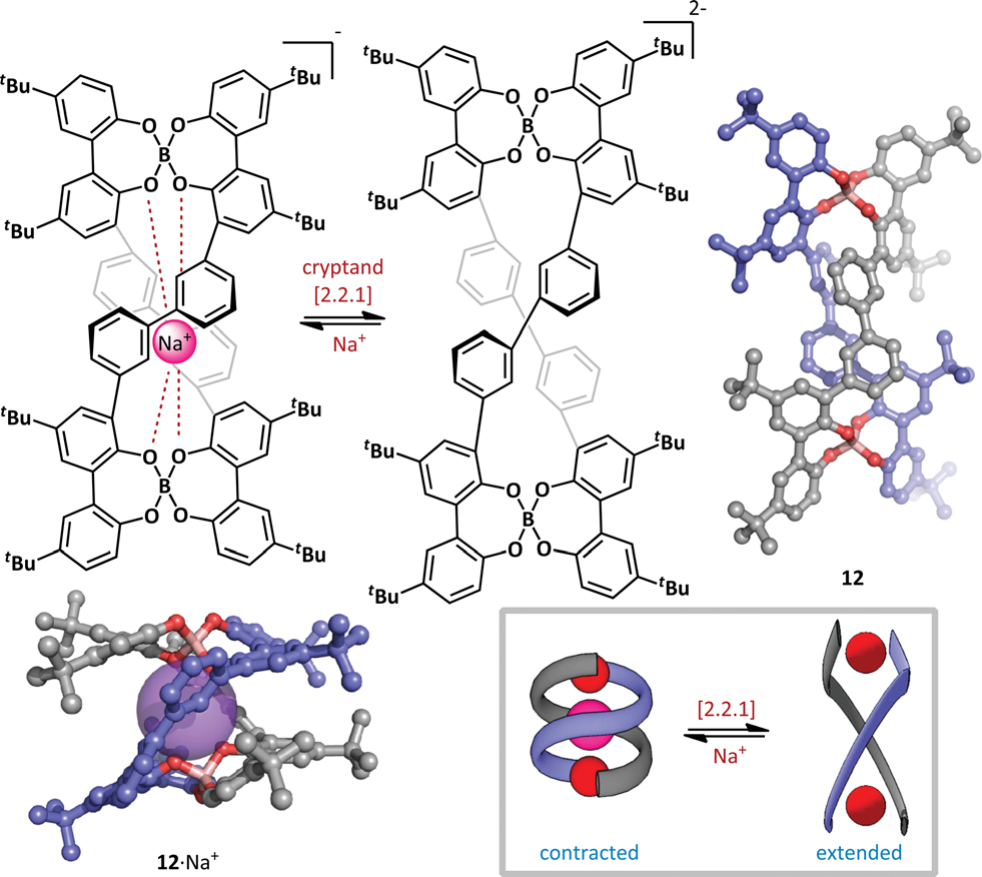
A chiral helicate **12** that undergoes conformational switching upon the addition or removal of sodium ions.

Furusho and Yashima have described a spring-like helicate molecular switch based on a tetraphenol boronate macrocyle (Scheme 10).^59^ The switch **12** was generated in an enantioenriched form (up to 93% ee) by treatment of the parent tetraphenol with sodium borohydride and subsequent resolution by co-crystallization with (–)-*N*-dodecyl-*N*-methylephedrinium bromide. The (*P*,*P*)-double helix thus formed efficiently complexes Na^+^ with the anionic boronates stabilizing the proximal cation, resulting in a B–B distance of just 6.0 Å and end-to-end twist of approximately 360°. Addition of a ten-fold excess of cryptand [2.2.1] efficiently removed the sodium ion and triggered a large conformational change in the double helix; the B–B distance is greatly increased (to 13.0 Å) and the curvature of the helix is reduced such that the twisting angle between helix termini is approximately 180°. The authors postulate that these changes are the result of electrostatic repulsion between the two boronate complexes upon removal of the sodium ion. The conformational change proceeds without loss of helical chirality, suggesting that switching occurs *via* a relaxation of intact macrocycle, rather than by fragmentation and recombination. Such an ion-driven chiral molecular piston has potential as the basis for future nanoscale molecular devices.

**Scheme 11:**
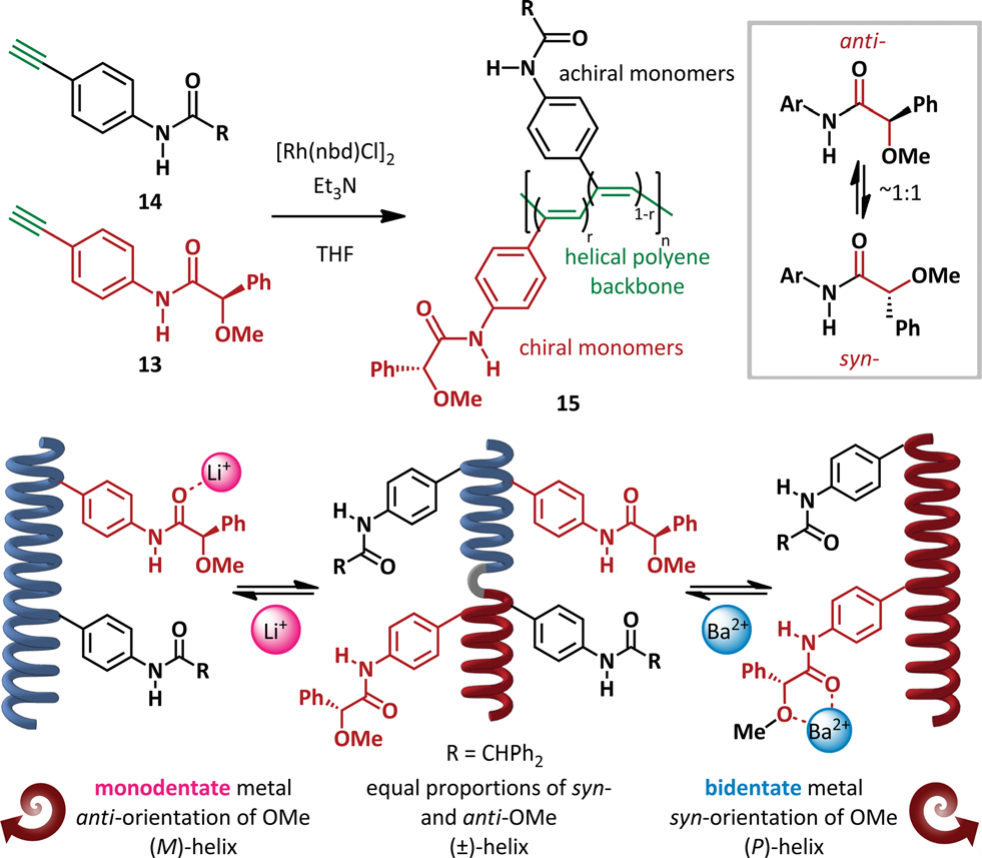
Selective metal-ion controlled formation of single helical *pseudo*-enantiomers: the sergeants and soldiers effect.

Freire and Riguera have recently outlined a novel approach to the ‘sergeants and soldiers’ concept of Green and Reidy (Scheme 11).^60,61^ In its classical form, this relates to the common effect that a chiral monomer ‘sergeant’ present in small amounts relative to the achiral ‘soldier’ monomers can have a disproportionate influence on the absolute helical chirality of polymeric chains. In the recent work, the sergeants are rendered switchable and only act when an external stimulus is applied. Rhodium-catalyzed copolymerization of chiral **13** and achiral **14** *para*-amidophenylacetylenes in a variety of ratios (chiral:achiral, *r*:(1–*r*)) generated *cis*-polyacetylenes **15** with molecular weights of 30–40 kDa. (*R*)-α-Methoxy-α-phenylacetic acid (MPA) was employed as the homochiral sergeant, chosen since it is known to equally occupy both *syn*- and *anti*-conformations. In the absence of any stimulus, the polymers behave as helical racemates. The *syn*- and *anti*-**13** monomers, present in equal quantities, favour (*P*)- and (*M*)-helices respectively to equal extents, with the combined effect of producing a *pseudo*-racemic mixture of helices. Upon the addition of a monodentate metal such as Li^+^, coordination to the amide favours the *anti*-conformation and concomitant formation of exclusively (*M*)-helices, as demonstrated by CD spectroscopy. Conversely, coordination to bidentate metals such as Ba^2+^ favours the *syn*-amide and hence the (*P*)-helical conformer. The critical importance of the amide geometry was demonstrated by DFT analysis, which verified the preference for (*M*)-helicity of a polymer consisting of achiral “soldiers” and a chiral *anti*-amide bound to a monodentate metal ion, and the converse for the *syn*-amide generated by bidentate metals. These switchable helices were subsequently demonstrated to be competent building blocks for tuneable nanospheres capable of encapsulating materials including iron oxide nanoparticles, quantum dots and fluorescent organic dyes.

## Anion binding

Although there are a number of examples of phenylacetylene and macrocylic based receptors for anions this area is significantly less well developed than cation binding.^62^ Typically these systems rely on hydrogen bonding as the primary effector of anion-induced conformational change, with intramolecular hydrogen bonds being replaced by intermolecular bonding to the host anion. In order for these intermolecular forces to compete with the native intramolecular fold, polydentate binding is typically employed; after formation of the first hydrogen bond to the anion, the entropic penalty for the formation of additional hydrogen bonds is greatly reduced.

### Macrocyclic systems

**Scheme 12:**
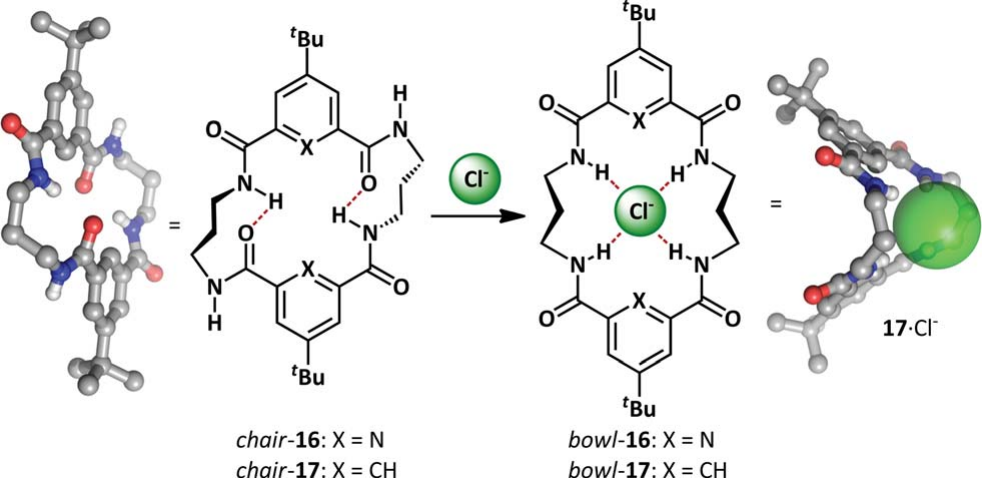
Conformational change upon Jurczak's macrocyclic polyamides **16** and **17** binding chloride.

As part of a research program exploring macrocycle anion-binding, the Jurczak group synthesized a series of macrocyclic amides that adopt a chair conformation and undergo an unfolding process upon anion addition (Scheme 12).^63–65^ Pyridine-2,6-dicarboxylate derivative **16** gave good binding selectivity between a range of anions including chloride, benzoate, acetate and dihydrogen phosphate. A related 20-membered cycle lacking the pyridine nitrogens **17** bound anions more weakly but had the advantage of allowing co-crystallization with chloride in the bowl conformation. Upon complexation both **16** and **17** exhibit conformational change from a closed, intramolecularly hydrogen-bonded macrocyclic chair to an open bowl conformer in which there is a convergent projection of hydrogen bond donors towards the host anion.

**Scheme 13:**
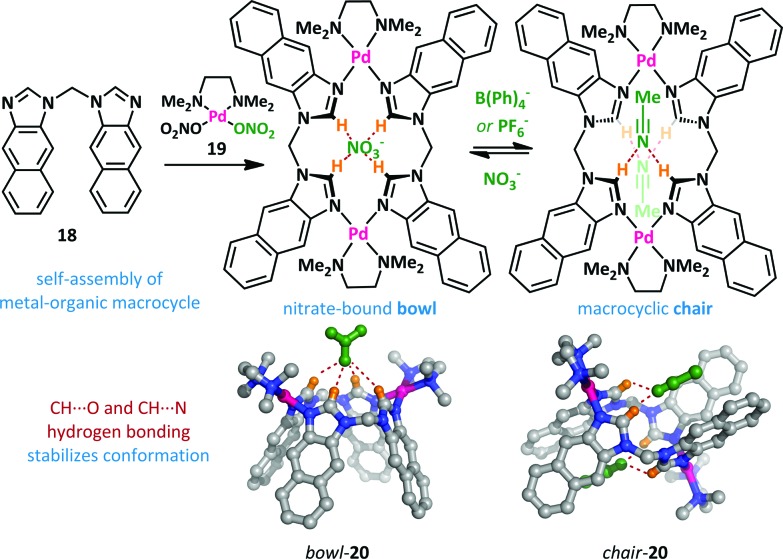
A C–H hydrogen bonding approach to anion binding leads to conformational switching between chair and bowl forms of **20**. For clarity non-coordinating counter-ions and solvents molecules are not illustrated.

Yu and Pan have reported a related macrocyclic approach to anion-mediated switching where, unusually, the hydrogen-bond stabilization of the anion is provided by multiple C–H donors (Scheme 13).^66,67^ When bis(imidazole) **18** is treated with one equivalent of palladium(ii) complex **19**, the nitrate complex **20** is formed quantitatively in a bowl-like conformation, with CH···O hydrogen bonding between the imidazolium hydrogens and the nitrate counter-ion providing the necessary enthalpic stabilization. However, upon addition of non-coordinating counter-ions such as PF_6_^–^ and B(Ph)_4_^–^, a large conformational change was observed. Partial inversion of the macrocycle occurs, allowing the formation of CH···N hydrogen bonds to acetonitrile, and reducing the steric clash between adjacent arenes.

### Phenylacetylene systems

The phenylacetylene motif is prevalent in the field of anion-mediated conformational switches. The motif is uniquely well-suited to the purpose since the inherent steric and electronic barriers to rotation are extremely low and interconversion between conformers is facile.^68^ By appropriate positioning of stabilizing groups such as hydrogen bond donor/acceptor pairs, conformations may be favoured thermodynamically and, since the kinetic barrier is low, the observed conformation will reflect the position of the equilibrium between rotamers.

**Scheme 14:**
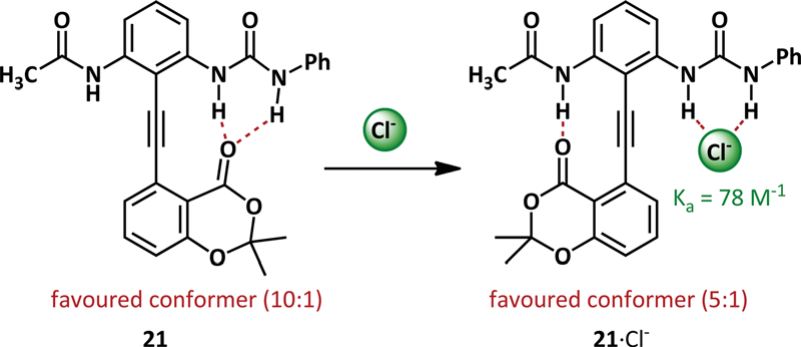
Urea-based anion binding by a diphenylacetylene scaffold **21**.

Within the Hamilton research group, a modification of the Kemp diphenylacetylene, **21**, has been used to probe anion binding (Scheme 14).^69^ In the absence of any stimulus the hydrogen bond acceptor lactone favours hydrogen bonding to the bidentate urea by 10 : 1. Upon complexation of chloride, the preferred conformation is switched, with the urea binding the chloride (with a binding constant of 78 M^–1^) and the lactone preferentially binds the acetamide.

**Scheme 15:**
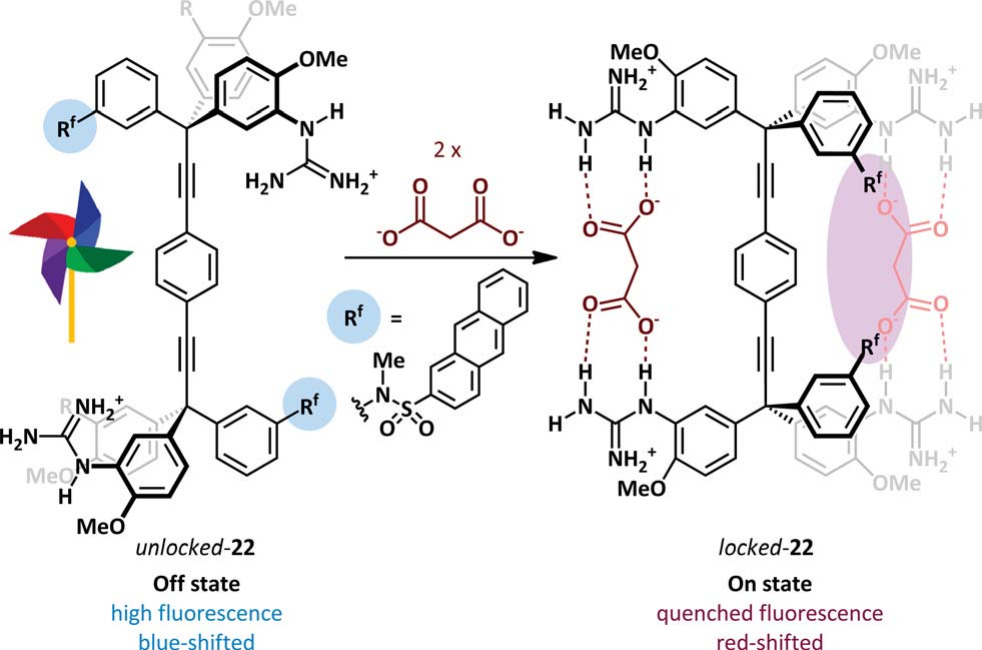
Dicarboxylate-induced conformational locking of a molecular pinwheel **22**.

The Glass group have described a ‘Pinwheel’ sensor **22** able to selectively detect dicarboxylates in aqueous solution with a fluorescent read-out.^70^ A bis-tritylacetylene scaffold supports four guanidinium recognition elements such that each pair may bind a dicarboxylate ion. Binding of the first dicarboxylate guest freezes rotation about the acetylenic bonds and thus preorganises the receptor for a cooperative second binding event (Hill coefficients^71^ of 1.7–2.0 for a range of dicarboxylates). The loss of rotational mobility positions two pendant anthracene sulfonanilidine groups adjacent to one another leading to a quenching of fluorescence (Scheme 15). A half-saturation value *K*_0.5_ – the concentration required to saturate 50% of the sensor in solution – of 1.96 × 10^–4^ M indicates that the sensor can quantify dicarboxylate concentration in the high micromolar range. Titration of switch **22** with large amounts of acetate did not give a response, while the addition of dicarboxylates in a solution containing 10 mM acetate gave only slightly lower binding constants than a solution lacking the competitive acetate guest, thus demonstrating excellent selectivity for dicarboxylate. For these pinwheel sensors the presence of the anionic stimulus slows molecular rotation, whereas Shimizu has described a related system in which binding of acetate increases rotational rate, forming the basis of a molecular rotor.^72^

**Scheme 16:**
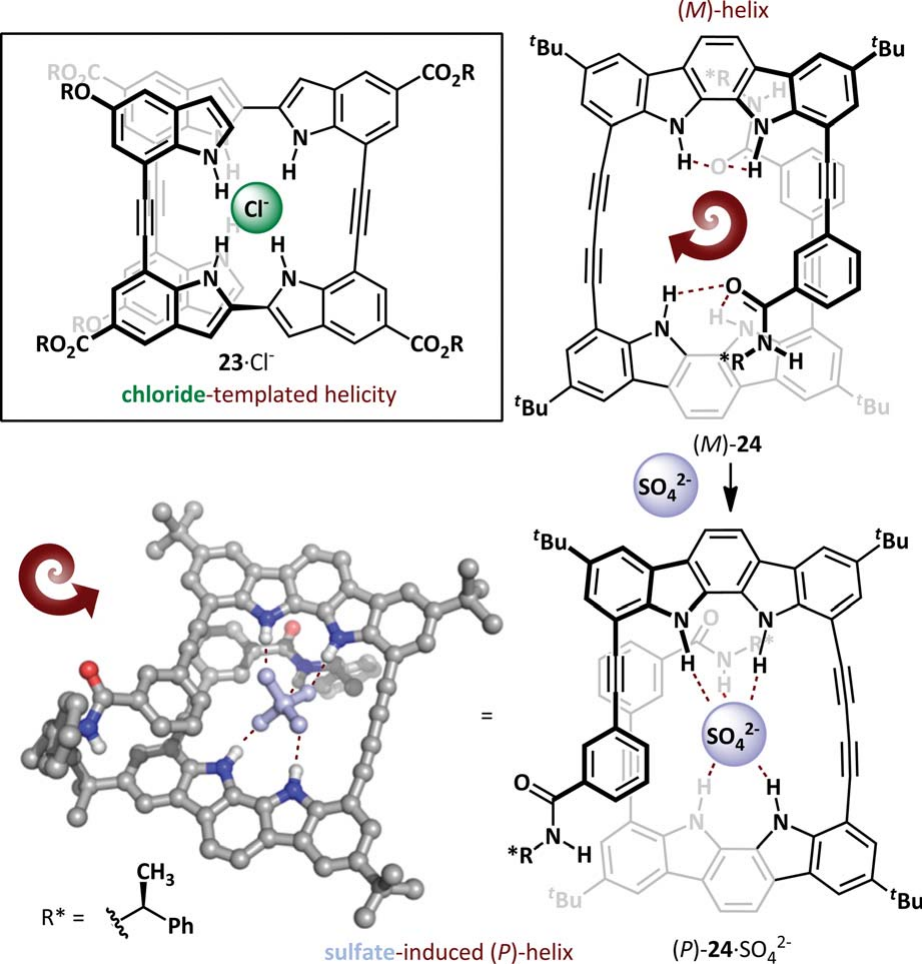
Helical switching in oligoindoles (**23**) and oligoindolocarbazoles (**24**) mediated by anion binding.

The Jeong research group used the diphenylacetylene linkage as the basis for a novel oligoindole foldamer **23** (Scheme 16).^73,74^ These adopt an unfolded conformation in the absence of stimuli, but exposure to chloride led to a helical conformation through N–H···Cl^–^ hydrogen bonding, with chloride binding constants of >10^7^ M^–1^ in acetonitrile. This concept has also been extended to oligoindolocarbazoles,^75,76^ where binding to a chiral organic sulfonate invokes a bias in the helical screw-sense. Introduction of a chiral directing group on the foldamer itself can also induce a bias towards (*P*)- or (*M*)-helical conformers^74,77^ and, remarkably, binding of an achiral sulfate anion can induce complete inversion of the preferred helical stereoisomer.^78^ Hecht has reported a spectacular host anion-dependent helical inversion in triazole-based foldamers; the native foldamer and fluoride adduct adopt the opposite screw-sense to the chloride, bromide and iodide adducts.^79^ A similar behaviour was reported by Jeong, wherein novel chiral indolocarbazole **24** was determined to adopt an (*M*)-helical conformation even in the absence of a coordinating anion, allowing weak out-of-plane intramolecular hydrogen bonding to occur between the indolocarbazole and chiral *N*-(α-methylbenzyl)amide. Exposure to bis(tetrabutylammonium)sulfate induces helical inversion, with concomitant changes in the ^1^H NMR (since the helices are diastereomeric) and CD spectra, with the latter exhibiting complete inversion and giving a positive Cotton effect for the sulfate complex, indicative of the formation of a (*P*)-helix. This was confirmed by X-ray crystallography, which demonstrated that the sulfate was engaged in hydrogen bonding with both the indolocarbazole and amide NHs. The fact that this switch has an optical readout (through inversion of the CD spectrum) bodes well for its use as the basis for chemoselective sensor applications.

## Conclusions

Extensive work during the last decade has led to the development of a broad toolkit of molecular architectures that switch conformation in response to a specific stimulus. Throughout this time numerous potential applications and opportunities for further miniaturisation of existing devices have been promised. Whilst there is scope to add new tools to our repertoire, arguably the greatest challenge for the next decade is their broader application in functional settings. With the exception of organic light emitting diodes and photovoltaics, the miniaturisation evident in our everyday lives is driven primarily by increased shrinkage of the silicon-based microchip, thus presenting plenty of opportunities for the use of single-molecule substitutes. As an example of targeted drug delivery, the antibody–drug conjugate in which a warhead is localised and delivered to a specific site is gaining traction as a viable therapeutic approach. Likewise photo-uncaging has attracted much recent attention, and so perhaps above all others medicinal goals will serve as the greatest motivation towards putting molecular switches to work. In this setting, rather than competing with existing technologies, there is an opportunity to deliver targeted therapy in an entirely new way.
